# Analyzing Cyber-Physical Threats on Robotic Platforms †

**DOI:** 10.3390/s18051643

**Published:** 2018-05-21

**Authors:** Khalil M. Ahmad Yousef, Anas AlMajali, Salah Abu Ghalyon, Waleed Dweik, Bassam J. Mohd

**Affiliations:** 1 Department of Computer Engineering, The Hashemite University, Zarqa 13115, Jordan; almajali@hu.edu.jo (A.A.); salah.g.ghalyon@hu.edu.jo (S.A.G.); bassam@hu.edu.jo (B.J.M.); 2 Department of Computer Engineering, The University of Jordan, Amman 11942, Jordan; w.dweik@ju.edu.jo

**Keywords:** mobile robot, robotic platform, cyber-physical systems, threats, security, risk management, risk assessment

## Abstract

Robots are increasingly involved in our daily lives. Fundamental to robots are the communication link (or stream) and the applications that connect the robots to their clients or users. Such communication link and applications are usually supported through client/server network connection. This networking system is amenable of being attacked and vulnerable to the security threats. Ensuring security and privacy for robotic platforms is thus critical, as failures and attacks could have devastating consequences. In this paper, we examine several cyber-physical security threats that are unique to the robotic platforms; specifically the communication link and the applications. Threats target integrity, availability and confidential security requirements of the robotic platforms, which use MobileEyes/arnlServer client/server applications. A robot attack tool (RAT) was developed to perform specific security attacks. An impact-oriented approach was adopted to analyze the assessment results of the attacks. Tests and experiments of attacks were conducted in simulation environment and physically on the robot. The simulation environment was based on MobileSim; a software tool for simulating, debugging and experimenting on MobileRobots/ActivMedia platforms and their environments. The robot platform PeopleBot^TM^ was used for physical experiments. The analysis and testing results show that certain attacks were successful at breaching the robot security. Integrity attacks modified commands and manipulated the robot behavior. Availability attacks were able to cause Denial-of-Service (DoS) and the robot was not responsive to MobileEyes commands. Integrity and availability attacks caused sensitive information on the robot to be hijacked. To mitigate security threats, we provide possible mitigation techniques and suggestions to raise awareness of threats on the robotic platforms, especially when the robots are involved in critical missions or applications.

## 1. Introduction

Nowadays, robots are increasingly involved in people’s daily lives, e.g., autonomous cars, drones, entertainment robots, etc. In fact, they become integral parts in different domains. For example, robotic vacuum cleaners are frequently used in our homes and households, Amazon Prime Air drones provide different services (e.g., pizza delivery), robots are being used in factory assembly lines (e.g., automobiles) and in medical settings, and, recently, autonomous cars are licensed in several states in the USA (e.g., Uber’s self-driving cars in Arizona, Pittsburgh and California [1]). Furthermore, the future of autonomous or semi-autonomous robotics in civilian, industry and military fields, and in almost every field in our living world could solve many of the problems and save human lives, power and time [2]. For example, military autonomous robotics are being used in both air and ground maneuvers [3].

Ensuring security and privacy or simply cyber-security for the robotic platforms is thus critical, as failures and attacks could have devastating consequences. For example, let us consider some possible security and privacy issues related to a robotic mapping and localization application (i.e., Simultaneous Localization And Mapping (SLAM) [4]). In such application, the robot would autonomously create a map of certain unknown environment and simultaneously localize itself in the constructed map. This requires bidirectional communication between the robot and the operator (user) to transmit commands and data. In a military application, these data are very important for performing surveillance and combat missions.

Considering the previous application, what if an attacker or unauthorized entity managed to successfully cause loss of integrity, availability or confidentiality on the robotic system (i.e., the robot, controller, and the communication link)? The attacker may exploit certain vulnerabilities in the robotic system to take control over the robot. Consequently, in the worst case, the attacker may crash the robot ending the military operation. Furthermore, the attacker may override the commands of the legitimate controller and possibly cause harm to civilians using the robot’s pre-installed military equipment. The previous attacks violate the integrity and availability of the robotic system. There may also be a situation wherein the attacker makes his/her way to sensitive information or records on the robot compromising confidentiality. In the worst case, the attacker may get the location of the robot or the operator compromising their physical safety. Additionally, the attacker may know other sensitive and classified information about the operator or the corresponding military facility, which can be used to perform a terrorist operation against the operator or the corresponding military facility.

Therefore, the cyber-security threats and consequences on the robotic platforms must be extensively studied and investigated. However, it is currently well-known that cyber-security for the robotic platforms is considered a big challenge because it requires an inter-disciplinary effort [5,6]. As such, this subject is currently a major challenge for robotic and autonomous systems. Different studies on the cyber-security of the robotic platforms generally target one or more of the following aspects: physical processes, computational resources, communication capabilities, operating systems, robotic libraries or middlewares, robotic applications and sensory information.

Majority of the robotic cyber-security studies have focused on the study and analysis of the security threats that target the robotic platform operating systems and their libraries/applications. For example, the main operating systems generally installed on a robot’s main computer are Microsoft Windows platforms (e.g., Windows 10 or Windows 7) and Linux open source platforms (e.g., Ubuntu). These operating systems require additional libraries and software that are robotic-specific to successfully operate different types of robots. It is well known that those operating systems have several known vulnerabilities [7,8,9]. Some of those vulnerabilities were recently analyzed in [10] for the PeopleBot robot. The authors implemented two denial-of-service (DoS) attacks, which rendered the robot unavailable.

One of the most popular studied robotic middleware for cyber-security threats is Robot Operating System (ROS), which is an open-source set of software libraries and tools for building robotics applications. ROS has several specialized branches including ROS-M, which is tailored to the unique needs of military robots. Additionally, there is ROS-INDUSTRIAL, which extends the advanced capabilities of ROS software to manufacturing. One fact about ROS is that it was designed without taking into account security protections, because it was mainly designed for research purposes. In fact, as reported in [5,11,12,13,14,15], several security flaws were found in ROS. Additionally, malicious malware can easily interfere with ROS communications, read ongoing messages or supersede ROS nodes. It is worth mentioning that a new version of ROS is under development (known as ROS 2.0 [16]) to solve the currently identified and reported security flaws.

The main objectives of this paper are to:

- Analyze several cyber-physical security threats, which target the communication link between Adept mobile robots research/academic platforms and their clients or users [17].
- Improve safety and security of the robotic platforms, raise awareness and increase understanding of the emerging threats on the robotic platforms.

To achieve the above objectives, we executed the following steps:

- Analyzed and utilized vulnerabilities in the communication link and other vulnerabilities that we discovered in MobileEyes/arnlServer client/server robotic applications. Those applications are necessary to establish the network connection between Adept mobile robots and their clients or users [18].
- Focused our analysis on possible security and privacy issues related to mapping and localization robotic scenario.
- Targeted three major security requirements, integrity, availability, and confidentiality, using an impact-oriented approach.
- Developed a novel robot attack tool (RAT) responsible for carrying out security attacks on integrity, availability, and confidentiality.
- Performed two risk assessment evaluation setups: a simulation-based approach using MobileSim simulator, and a real-life evaluation using the PeopleBot^TM^ [17]. The risk level of threats were qualitatively assessed (i.e., low, moderate, and high).
- Identified possible physical consequences from integrity, availability, and confidentiality attacks.
- Suggested numerous mitigation techniques to reduce or prevent the risk of attacks.

The rest of the paper is organized as follows. In Section 2, related work is discussed. In Section 3, we introduce the mobile robot platform under study, the PeopleBot, and the applications used to connect to the robot. In Section 4, we present the risk assessment approach used in this paper, our developed robot attack tool (RAT), and discussion on the performed security attacks (their physical impacts and some of the mitigation techniques). Finally, we conclude the paper and provide our future directions in Section 5.

## 2. Related Work

Recently, the studies on the cyber-security aspects relate to the robotic platforms or cyber-physical systems (CPSs) have received extensive attention by the research community with numerous published articles and Special Issues targeting this subject (e.g., Special Issue on cyber-security in robotics and autonomous systems in Elsevier [6] and Special Issue on Safety, Security, and Rescue Robotics (SSRR) in the journal of Field Robotics in Wiley). Most of the studies focused on how to have a safer and more secure robotic systems by addressing one or more of the following issues:

- identification of possible security and privacy threats against many modalities of robotic systems;
- evaluation of impact of the identified threats in a systematic manner; and
- prevention and mitigation of the threats.

The answers to the aforementioned issues altogether are known in the literature as security risk assessment. In fact, the security risk assessment on the robotic platforms is an active and rapidly growing research area due to its increasing significance. Despite that, the number of publications addressing risk assessment on the robotic platforms is limited, and several articles stated that security risk assessment tends to be an overlooked issue in robotic systems [15]. The main reason is that the focus is commonly placed in robot functionality, design and innovation. Further, there is inadequate understanding of what are the actual risks and the affected security goals [19]. Next, we present a thorough summary from literature about some of the studies that targeted security threat analysis and detection for several robot platforms or CPSs.

Martín et al. [20] analyzed the quality of different communication solutions used in robotics, when security features are enabled. The authors analysis focused on robotic applications that are based on distributed middleware and transmitted regular robotic data packets of different sizes on various network settings. The analysis results showed that the communication quality in terms of latency and packet loss rate is acceptable; hence, the authors recommended that security capabilities should be enabled in common cases. Sabaliauskaite et al. [21] proposed an experimental approach to assess the efficacy of cyber-attack detection methods when applied to robotic platforms. To evaluate their approach, the authors used the educational robots, AmigoBots^TM^, and applied three cyber-attacks (i.e., injection, stealthy, and scaling) and seven sensor attack detection methods. The evaluation results indicate that M1 and M5 are most effective for detecting cyber-attacks on AmigoBots.

Breiling et al. [14] presented a secure communication channel that enables ROS-nodes to communicate with authenticity and confidentiality using cryptographic methods (Transport Layer Security (TLS) and Datagram Transport Layer Security (DTLS)). The authors described the implementation changes to ROS core and assessed the overhead introduced by embedding the new security functions. They provided their implementation source code to the Open Source Robotics Foundation (OSRF) for consideration to be included in the SROS project [22] and ROS 2.0. In [23], Dieber et al. presented the most common security vulnerabilities of ROS and gave examples of attack vectors that can easily exploit vulnerabilities. Moreover, the authors proposed an application-level approach that helps to secure small ROS applications by securing all communication channels without being invasive to the ROS source code. Abeykoon and Feng [13] performed a formalized and structured forensic investigation of ROS. They focused on creating a formalized and analytical framework to acquire related digital evidence after a forensic investigation of cyber-crimes such as that of ROS.

Guerrero et al. [24] focused on cyber-security attacks that target Real Time Location Systems (RTLSs), which are critical components for many robots and autonomous systems. The authors showed that such attacks can be detected by machine and supervised learning techniques. Different learning algorithms were analyzed using real data collected by a wheeled robot equipped with a RTLS based on ultra wideband beacons. The experimental results indicated that the Multi-Layer Perceptron classifier is most effective for detecting DoS and spoofing cyber-attacks on RTLSs. Shrivastava et al. [25] proposed an orthogonal defense mechanism (ODM) to detect cyber-attacks on an operational water treatment plant. ODM stealthily monitors the plant using an independent network to collect data from various sources and corroborates the plant state. The proposed mechanism is orthogonal to any other defense mechanism that may exist. The design of the proposed ODM is generic and could be easily adapted for other CPSs.

Bezemskij et al. [26] presented a real-time sensor-agnostic methodology that runs on an onboard resource-constrained autonomous vehicle to detect different first-time cyber-physical and physical-cyber attacks. Their approach is based on formulating the detection method as a binary classification problem. In [27], Vuong et al. developed an intrusion detection system based on decision trees against DoS and Command Injection attacks. Most recently, Vuong et al. [28] presented two attack detection techniques for robotic vehicles. The first technique implemented detection using decision trees and the second technique utilized deep learning to efficiently detect various attacks. Similar to the second technique, Jones and Straub [29] designed a two-stage intrusion detection system (IDS) to reveal the existence of intrusions and malware in autonomous robots. A deep neural network is trained to detect behavior deviations.

Javaid et al. [30] investigated multiple security threats of Unmanned Aerial Vehicles (UAV) by listing vulnerabilities, which can be exploited to negatively impact confidentiality, integrity, and availability. The risk level of an attack was evaluated by multiplying the estimated likelihood and the attack impact. Batson et al. [31] identified threats and vulnerabilities in the system’s concept for Unmanned Tactical Autonomous Control and Collaboration (UTACC). Quarta et al. [32] experimentally analyzed the security of an ABB 6-axis IRB140/IRC5 industrial robot controller. Several software vulnerabilities in the robot main computer were exposed. These vulnerabilities are associated with the network services necessary for the robot’s operation (e.g., FTP service).

Bonaci et al. [33] considered and studied the types of attacks that might occur (e.g., the robot is taken over and turned into a weapon) and their implications on rescue and recovery missions. More recently, Bonaci et al. [19] analyzed the security threats for the Raven II robot, which is teleoperated for surgical purposes. The analysis showed that many of the robot tasks can be maliciously altered using manipulation and disruption attacks on the wireless communication link between the robot and the user (i.e., the surgeon). The implemented attacks were based on the man-in-the-middle model and they negatively impacted the usability and the safety of the robot, which might result in privacy and legal violations. Similarly, Alemzadeh et al. [34] demonstrated targeted cyber-physical attacks on teleoperated surgical robots, by exploiting vulnerabilities in the robot’s control system. They showed that such attacks can lead to catastrophic consequences in the physical system (RAVEN II robotic arm) and/or patient. Alemzadeh et al. presented a model-based analysis framework to estimate the consequences of control commands within the real-time constraints of the control system or robot’s dynamics.

Chen et al. [35] modeled and studied integrity attacks against CPSs such as automobiles and robotic platforms. They formulated a quadratic cost function that captures the attacker’s control objectives to avoid detection. Clark et al. [36] identified current and potential cyber-attacks to robotics at different levels; firmware/OS and hardware. Possible attack scenarios at each level were presented and discussed. Additionally, possible mitigation techniques were suggested. Deng et al. [37] used the 3GPP confidentiality and integrity algorithms (f9 specification) to protect the integrity of robot’s remote information. They used FPGA to realize the hard core algorithm KASUMI of the f9, and gave an implementation example.

Guiochet et al. [38] adapted a classic risk assessment approach applied during the initial phases of the development process for autonomous systems such as service robots. Initially, the authors used Unified Modeling Language (UML) to model tasks and preliminary analyze application domain hazards. Then, during the specification phase, a risk assessment of the robotic system was performed. The analysis is based on the guideword-based collaborative method HAZOP (HAZard OPerability) applied to UML models. The proposed risk assessment approach was applied on an assistive robot providing assistance for standing up, sitting down and walking, and health-state monitoring. Khalid et al. [39] identified the basic technology and functional requirements of a cyber-physical system to control human–robot collaboration (HRC) in an industrial context. Various safety approaches for heavy payload robots were discussed. The authors concluded with a general guideline that was formulated to serve for industrial level HRC scenarios. More recently, Khalid et al. [40] introduced a security framework for the application of HRC. They identified the basic elements and functional requirements of a secure collaborative robotic CPS. The impact of their proposed framework is demonstrated on a teleoperation benchmark (NeCS-Car).

Portugal et al. [15] discussed the cyber-security and cyber-safety issues in human–robot shared environments. They focused on surveying existing work and analyzing the security issues in ROS-based systems. Kriaa et al. [41] provided a comprehensive survey of existing design and risk assessment studies that consider both security and safety for industrial infrastructures. McLean et al. [42] described a method for identifying the risks involved with mobile agent systems through a “Risk Management” approach. They also provided and described implementations of security features relevant to such systems. Vuong et al. [43] investigated physical indicators of cyber-attacks on a rescue robot, which can adversely affect its operation and impair an emergency response action. Their approach is based on DoS attack launched against the robotic vehicle. Wardzinski [44] proposed a model for an autonomous vehicle control system, which utilizes risk assessment of the current and foreseen situations to plan its movement at an acceptable risk level.

Chowdhury et al. [45] presented a survey of recent cyber-security attacks on robotic systems, including: DoS attacks, extortapalooza public shaming and extortion attacks, impersonation attack, and sybil attacks. The authors discussed the possible economics losses due to these attacks and suggested mitigation approaches. Priyadarshini [2] explored the necessity of cyber-security in robotics. She discussed several case studies of different kinds of robots used in different fields (e.g., WowWee Rovio, Erector Spykee, and WowWee RoboSapien V2), and showed that the current state of robotics is vulnerable to many risks. Mitigation strategies were discussed at the end to avoid cyber-security risks. Matellan et al. [46] focused on the importance of cyber-security in the robotics platforms in general, and concluded that the protection of robots from cyber-attacks is not fully assured.

This paper is an extension to our previous paper [10] in which we focused on vulnerabilities of the operating system running on the robot and the client. Those vulnerabilities are not unique to the robot and may apply to any environment running the same configuration. However, this paper extends the work of [10] in the following ways:

- It presents a thorough summary from literature about the studies, which targeted security threat analysis and detection for several robot platforms or CPSs.
- It analyzes three different security requirements of the robot: integrity, availability and confidentiality. In contrast, the prior work only analyzed the availability security requirement.
- It exploits vulnerabilities in the robotic software that are unique to the robotic platforms; we analyze the cyber-physical security threats on the robotic platforms that specifically use the MobileEyes/arnlServer client/server applications, and we do not only exploit general vulnerabilities in operating systems that actually run on the robotic platforms (the prior work).
- While in [10] the security risk assessment was only performed physically on the robot, in this paper, we extend that by performing the assessment both physically on the robot and in simulation using MobileSim robot simulator.
- In this paper, we develop a robot attack tool (RAT) to perform several attacks on the client/robot communication stream, which we provided to the community as an open source code [47].

Our contributions to the state-of-the-art are summarized as follows:

- We analyze three different security objectives of the robot: integrity, availability and confidentiality.
- We apply a systematic methodology for risk assessment based on NIST adversarial risk assessment template proposed in [48].
- We develop a robot attack tool (RAT) to perform several attacks on the client/robot communication stream. This affects the integrity and availability security requirements of the robot.
- We provide RAT tool to the community as an open source code [47].
- We identify weaknesses and vulnerabilities in the robotic software (MobileEyes/arnlServer). To the best of our knowledge, this is the first study to address vulnerabilities in the robotic software of Adept MobileRobots platforms.

## 3. Robotic Platform and Software

### 3.1. The Robot Platform

**Figure 1:**
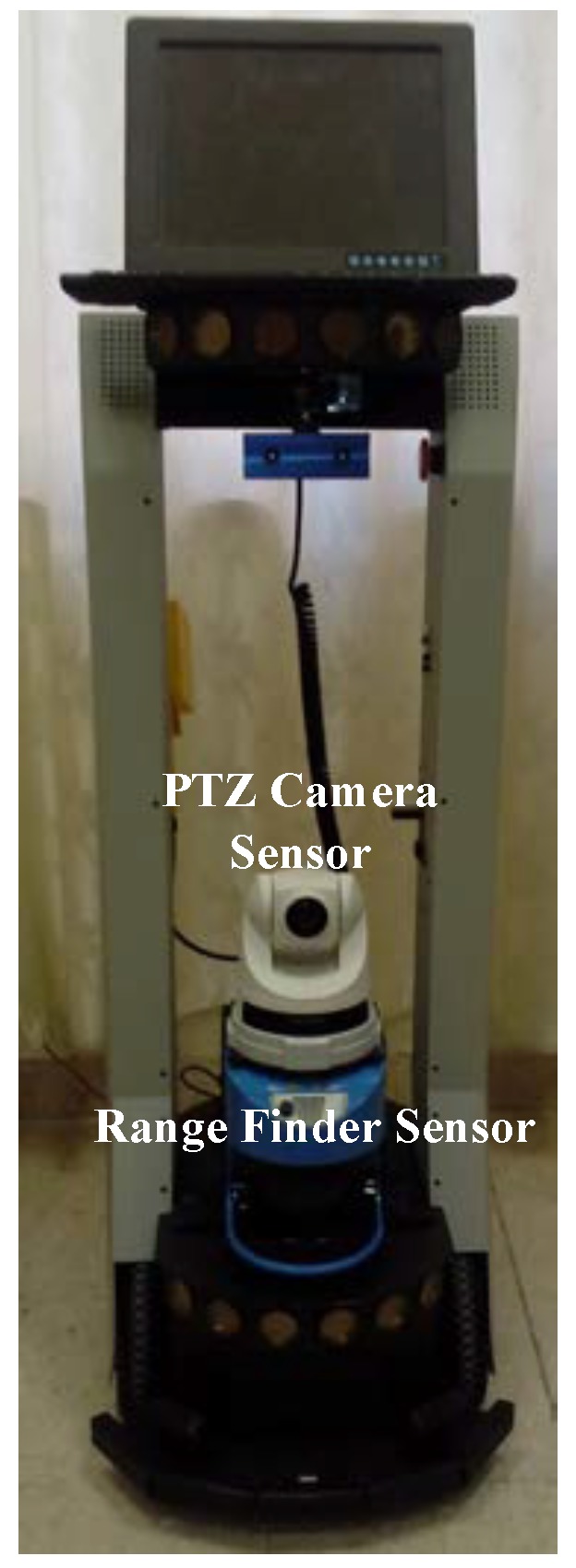
The PeopleBot mobile robot platform, where several sensors are attached to the robot, e.g., pan-tilt-zoom (PTZ) camera, laser range finder, sonars, stereo camera, etc.

The security risk assessment study presented in this paper specifically applies to the robotic platforms that use the MobileEyes/arnlServer client/server applications [49]. Such platforms are from Adept MobileRobots LLC incorporation [17]. We chose the PeopleBot mobile robot platform as a case study for our risk assessment analysis. Figure 1 shows the PeopleBot mobile robot platform equipped with several sensors (e.g., pan-tilt-zoom (PTZ) camera and laser range finder). The MobileEyes and arnlServer software are used to establish a communication channel between the robot and the operating user. In the case a physical robot is not available, the MobileSim simulator can be used to emulate the robot. This simulator supports several types of Adept robots. In the simulation, MobileEyes/arnlServer applications can be integrated with MobileSim such that to emulate establishing a link and controlling a real robot. In what follows, we briefly describe these applications, their usage, and the vulnerabilities.

#### 3.1.1. MobileEyes

**Figure 2:**
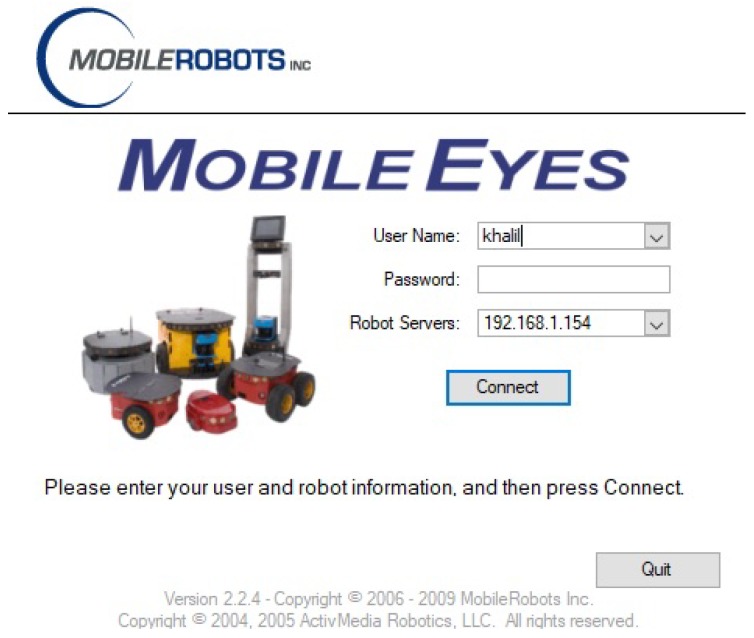
MobileEyes graphical user interface.

MobileEyes is a development software as well as a graphical user interface (GUI) used to remotely observe and control virtual or physical robots [18]. It has the ability to interact with a wide range of systems including the robot’s main computer and network connected Linux and Windows computers. MobileEyes uses a networking protocol, namely ArNetworking, to connect over a network to an ArNetworking-based server (e.g., ARNL’s guiServer, ArNetworking, serverDemo, arnlServer, and among others [50]). Using the ArNetworking protocol, MobileEyes communicates with the server via the TCP/IP networking protocol, port 7272. After startup, MobileEyes user locates the robot server by entering a hostname (e.g., “myRobot”) or an IP address (e.g., “10.239.40.170”) in the “Robot Servers” field in the MobileEyes’ GUI, as shown in Figure 2.

It is noteworthy that not all robot servers (e.g., arnlServer) are configured to require login credentials. In other words, by default, either one or both of the “User Name” and “Password” fields in Figure 2 can be left blank [51], without affecting the operation of MobileEyes. Those fields will only be required and useful if the robot server has been configured to require login credentials. In this case, the password will get authenticated using MD5 hash coding scheme, but the username will only get matched as a plain-text (without being first encrypted and then decrypted) in the traffic stream between the client (i.e., MobileEyes) and the server.

**Figure 3:**
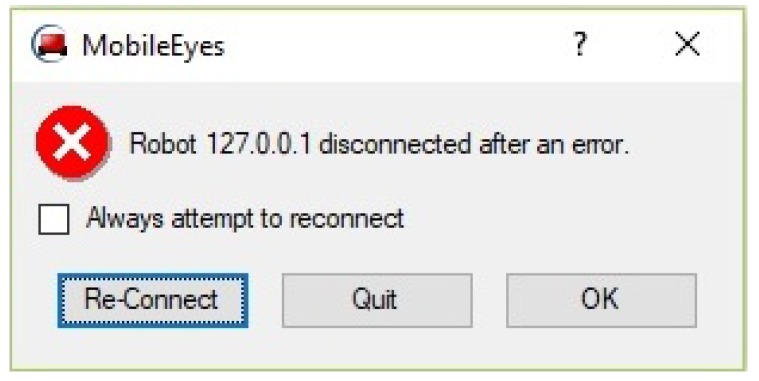
MobileEyes reconnect message if it loses connection during a session.

After entering the “Robot Servers” hostname, the user clicks the “Connect” button to access the robot over the network. The connection to the server that is running on the robot may then be canceled by clicking the “Quit” button. If MobileEyes cannot connect with the robot after a few seconds, it may show a message of why the connection failed and then try to reconnect again. One feature of MobileEye is that if it loses connection during a session with the server, it will give a message and an option to reconnect without having to stop and restart the GUI as shown in Figure 3. This feature is useful because it makes MobileEyes easier to use; however, it can be utilized by an attacker as a vulnerability or possible threat (e.g., in a man-in-the-middle (MITM) attack).

**Figure 4:**
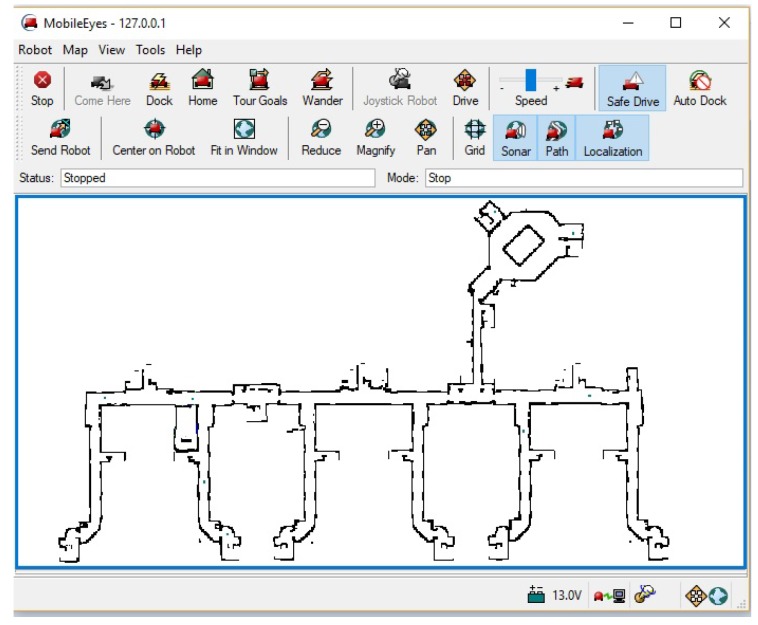
MobileEyes GUI after a successful connection to the server. A logged 2D laser map of the indoor hallways of the second floor of our engineering building is being loaded in the map pane of MobileEyes.

**Figure 5:**
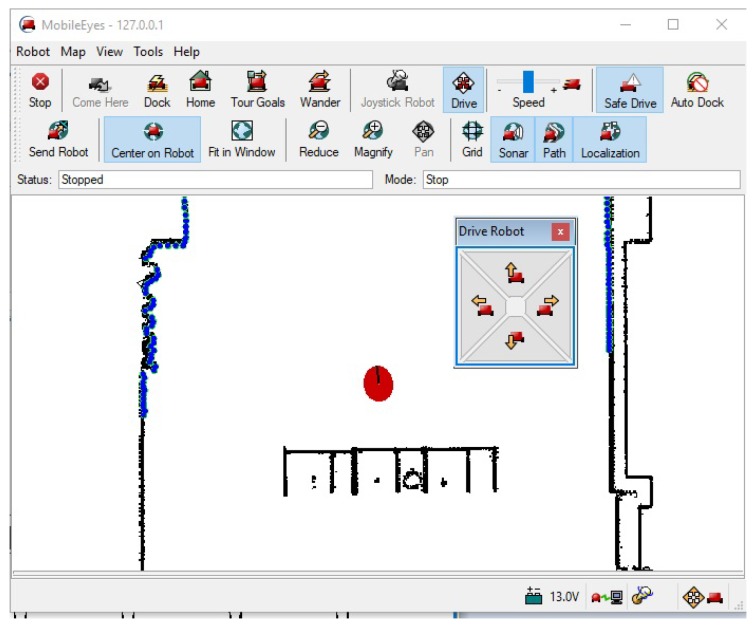
A zoomed view of MobileEyes GUI after a successful connection to the server showing the robot (red circle or icon in the middle of the map pane), its lidar data in blue colored points at the thickest lines in the pane, and the teleoperated window to drive the robot.

On a successful connection between MobileEyes and the server, and depending on what services the server has been configured to provide (access to camera(s), lidar(s), sonars, bumpers, IR’s, battery information, wheel encoders, etc.), MobileEyes can display the robot’s position in a map, range sensor (sonar, laser) data, camera image, and much more. The control features of MobileEyes GUI allow for teleoperating the robot and sending it to a goal in a loaded map of a certain environment, among many other useful features. Figure 4 and Figure 5 show two snapshots of the MobileEyes GUI after a successful connection to arnlServer.

In summary, from a security point of view, the following features of MobileEyes can be considered as possible threats on the robot:

- The port number is published and known. Thus, using any of the packet sniffing tools on the robot network, an attacker can determine the existence of the robot connection on the network by looking for port 7272.
- By default, the user does not need to specify a user or password to connect to the server, which can allow an attacker to login and connect to the robot by accessing the network and knowing the robot IP address.
- Even if the robot server is configured to require login credentials, the username information gets authenticated with the server as a plain-text without being encrypted. Thus, an attacker can easily figure out such information by packet sniffing the traffic stream between MobileEyes and the server. Additionally, the password get authenticated using MD5 hash coding scheme, which suffers from extensive vulnerabilities [52,53].
- If MobileEyes loses connection during a session, it can be configured using the pop-up dialog displayed in Figure 3 to reconnect automatically. Consequently, this allows an attacker to perform a MITM attack on the robot without being disconnected from the client side.

#### 3.1.2. arnlServer

arnlServer is an ArNetworking-based open source server with several APIs (C++, JAVA, and Python). It uses the ArNetworking protocol, briefly introduced above, to connect with MobileEyes or other remote control clients. arnlServer is provided as part of the Advanced Robotics Navigation and Localization (ARNL) library [50], which is used for intelligent navigation and localization.

ARNL comes in two packages: ARNL and BaseARNL. BaseARNL includes a software called Advanced Robotics Interface for Applications (ARIA) that is necessary to communicate with robots in addition to many other useful functions. In addition, ARIA includes the ArNetworking implementation. One application of arnlServer is indoor navigation using a laser range finder (LRF) sensor (i.e., a lidar), which is installed on the robot to map and perform precise localization; a technique known as Simultaneous Localization And Mapping (SLAM). To use arnlServer, at least three software distributions must be installed; MobileSim, MobileEyes and ARNL. If the user is dealing with a real robot, MobileSim will not be needed.

arnlServer is usually configured to enable/disable access to many features on the robot such as: laser data, sonar data, camera images, robot position, etc. This enables MobileEyes or other clients to monitor and control access to the robot and its accessories as mentioned above. To do so, arnlServer uses parameter files that are associated with both ARNL and the operating characteristics of the connected robot and its accessories. To override the default parameter files, arnlServer takes several arguments when it starts to specify and set new values. Additionally, there are options in the connected clients, such as MobileEyes, to send commands to arnlServer to set operating parameters.

Finally, arnlServer requires a map similar to the one shown in Figure 4. Such map is needed to define the robot’s operating space to perform the localization and navigation tasks on the onboard computer or remotely on another computer.

The vulnerabilities discovered in arnlServer are as follows:

- By default, the server is not configured to require the user (or client) to specify a username or password to connect to it. An attacker could potentially login and connect to the robot when accessing the robot network and providing the robot IP address.
- Even if the robot server is configured to require login credentials, the username information is sent as a plain-text without being encrypted, and the password is hashed using vulnerable MD5 hash coding scheme and sent over the network to the server.
- If the client connection with the server gets lost, say, due to bad network connection, then it will be restored automatically once the connection stream becomes okay without the need to explicitly reconnect again. Thus, if an attacker (e.g., in a MITM attack) interrupts the connection between the server (robot) and client for some time, then this might look like a bad network connection without anything noticed or any action taken from the server and the client.
- Most of the information sent from the server side to the client (i.e., robot position, laser data, and camera images) is sent in plain-text. Thus, a possible integrity attack might manipulate such information and result in serious damage to the physical robot and the surrounding environment.

### 3.2. The MobileSim Simulation Environment

MobileSim is a software used for simulating mobile robots and their environments [54]. It can be used for debugging and experimenting the software (e.g., MobileEyes and arnlServer) that support Adept mobile robots platforms when a real physical robot is not available. MobileSim is built upon the Stage simulator, which is part of the Player/Stage project [55].

MobileSim uses the robot map (similar to the one shown in Figure 4) to simulate walls and other obstacles in the environment. Additionally, it places a simulated robot model in that environment, and provides a simulated control connection accessible via a TCP port 8101. All communications with the simulated robot must occur on this specified TCP socket connection or channel. MobileSim supports many device models of MobileRobots/ActivMedia mobile robot bases (e.g., PowerBot^TM^, AmigoBot^TM^, and PeopleBot^TM^) along with many of their accessories (e.g., laser and sonar).

## 4. Risk Assessment of the Robotic Platform

Risk assessment is an essential part of the risk management process. Usually, risk management starts by assessing the risk. However, risk assessment is a continuous process that should be maintained during system development. Risk itself is function of vulnerability, threat and impact (harm) of an adverse event (a successful cyber-physical attack in this case). Vulnerabilities and threats indicate the likelihood of the adverse event [48]. In this paper, we are interested in the impacts of cyber-physical attacks on the functionality of the robotic platform. Hence, an impact-oriented analysis is adopted and only vulnerabilities that lead to those impacts are investigated. That is, we only analyze the vulnerabilities that may lead to harmful cyber-physical impacts on the robot.

### 4.1. Attack Analysis

As stated earlier, many applications operate robots remotely. In our case, a user can connect to a robot in a client–server mode using MobileEyes and arnlServer. Any loss in the integrity or the availability of the communication results in negative cyber-physical impacts [40,56]. In the following, we first present an analysis for some of the integrity attacks followed by an analysis for some of the availability attacks that are all specific to the communication stream between MobileEyes and arnlServer. Then, we briefly comment on the confidentiality aspects of the robot that can result from the performed integrity or availability attacks.

#### 4.1.1. Analysis of Integrity Attacks

**Figure 6:**
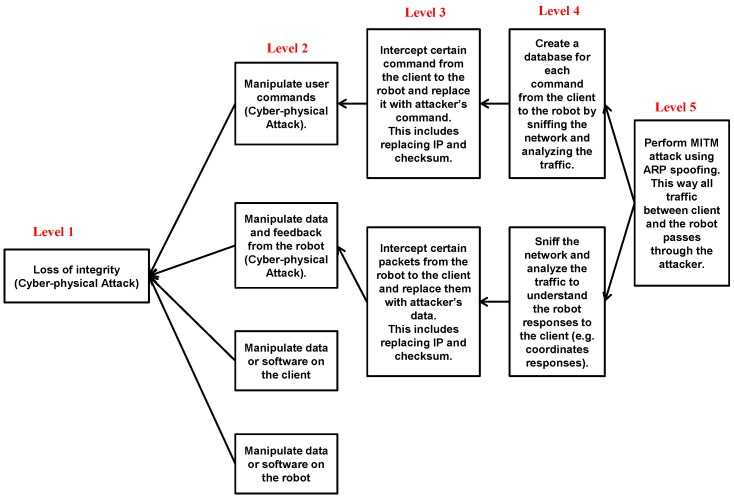
Integrity attack tree.

A good representative example of an integrity attack would be if a user is teleoperating a robot using MobileEyes (see Figure 5) and wants the robot to turn left, but a MITM attacker intercepts this command and forces the robot to turn right instead. In this case, there is a chance of physical damage to occur including falls or collisions. Other integrity scenarios are also possible. In Figure 6, we show an attack tree that demonstrates different branches of attacks based on a MITM attacker that can lead to the loss of integrity, which represents the ultimate objective of the attacker as demonstrated by Level 1 of the attack tree. The loss of integrity is achieved by manipulating the communication stream between the client (user using MobileEyes) and the server (robot) to cause cyber-physical impacts. More details on the cyber-physical impacts or consequences are given in Section 4.3. The branches of the tree represent the ways and techniques through which the attacker can achieve the ultimate goal. The arrows in the attack tree indicate the sequence through which each attack proceeds. The presented attack tree is not inclusive, meaning it does not cover all possible ways to achieve loss of integrity.

There are four main ways to perform an integrity attack as shown in Level 2 of the attack tree:

- Manipulate the traffic from the client to the robot.
- Manipulate the traffic from the robot to the client.
- Manipulate the data or software on the client.
- Manipulate the data or software on the robot.

Level 3 of the attack tree represents the traffic manipulation *techniques* that are used to achieve the consequences in Level 2. In this paper, for our assessment, we focus on and implement the first and second manipulations from the list above. The technique to achieve the first manipulation is based on intercepting the traffic that goes from the client to the robot, while the technique to achieve the second manipulation is based on intercepting the traffic that goes from the robot to the client. The third and fourth manipulations can be implemented using special malware, such as a computer virus or worm, but are beyond the scope of this paper.

Most of the traffic sent from the client to the robot are commands; however, most of the traffic sent from the robot to the client are data and feedback of certain tasks. Both integrity attacks in the tree start by performing a MITM attack through Address Resolution Protocol (ARP) spoofing on the robot and the client as indicated by Level 5. This requires the attacker to be on the same network as the robot and the client. In other words, the attacker is either a trusted insider or an outsider who managed to hack into the network. Level 4 of the attack tree shows that the attacker can analyze the packets and distinguish certain commands (e.g., movement commands) and data (e.g., coordinates and responses) by sniffing the traffic between the robot and the client.

To assess the risks associated with the two integrity manipulation threats considered in this paper, a python-based robot attack tool (RAT) that utilizes Scapy [57] is developed. To use the RAT tool, MITM attack must first be successfully initiated and mounted on the robot network. Thus, this tool is supposed to run on the MITM attacker machine to perform the intended attacks. RAT automatically intercepts certain commands (in the form of TCP packets) that are transmitted from the client to the robot and replaces them by commands of our choice; namely, this is known as a replay attack. The delay introduced into the communication link by using the RAT tool is negligible and unnoticeable for the user (usually less than 0.75 s). For example, RAT can force the robot to turn left instead of turning right as directed by the client. This is achieved by manipulating the headers of the packets, such as changing the source and destination IPs. By the same token, traffic from the robot to the client can be manipulated. The robot may send its coordinates or the readings of certain sensors to the client, but these data can be replaced by data of the attacker’s choice using RAT.

#### 4.1.2. Analysis of Availability Attacks

**Figure 7:**
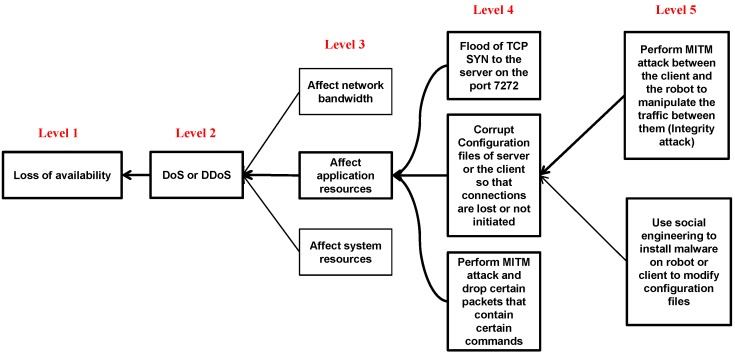
Availability attack tree.

Figure 7 represents the attack tree of the loss of availability. In this paper, the focus is on analyzing attacks targeting vulnerabilities specific to the robot system applications, as indicated by the rectangles with the bold borders in Level 3. Attacking network bandwidth and system resources are not discussed in this paper as they were fully covered in our previous work [10].

The first (i.e., upper) path from Level 4 in the attack tree in Figure 7, which affects the application resources, exploits the fact that port 7272 is always open on the robot. The client (MobileEyes) uses this port to connect to arnlServer, as mentioned in Section 3. This can be used by an attacker to implement a TCP SYN flood by sending a TCP SYN requests to the robot (server in this case) and not responding to the SYN-ACK of the robot. This consumes robot’s resources and legitimate traffic gets lost making the robot unavailable to the client. The cyber-physical consequences of having the robot unavailable are discussed in Section 4.3.

The second (i.e., middle) path from Level 4 in the attack tree, which causes loss of availability relies on RAT. First, a MITM attack is used to manipulate the traffic. As long as the attacker can intercept the traffic between the robot and the client, he/she can send modified commands to change configuration files (i.e., parameter files mentioned in Section 3.1.2) on the robot and the client. Alternatively, the attacker can install a malware or a virus on the robot or the client to overwrite the parameter files. In the last (i.e., lower) attack path from Level 4 in Figure 7, the attacker can also use a tool like RAT to drop all or some of the commands between the robot and the client, so that functions of the attacker’s choice are rendered unavailable.

The algorithmic steps for both the integrity and availability attacks, which are performed through the RAT tool, are shown in Algorithm 1. Prior to running the RAT, an attacker should be able to successfully perform a MITM attack. After which, the RAT tool sniffs the packets streamed over the network. Based on the direction of the traffic (robot to client and client to robot ), the RAT tool accepts five states that indicate the type of the attack, which are assigned to a variable named as **State**. The first fours states represent integrity attacks, and the last one represents an availability attack. These states are described as follows:

- States of integrity attacks:
  - mirror: Reverse the direction of the movement of the robot; change right command to left, and left command to right.
  - send_to_position: Replace any command by a command that forces the robot to go to a certain position of the attacker’s choice.
  - circulate: Replace any command by a command that forces the robot to circulate in one direction (left).
  - fake_position: Modify robot position sent to the client.
- State of an availability attack:
  - availability_attack: Drop the packets in any direction between the client and the robot rendering the robot unavailable.

**Algorithm 1** MITM Algorithm Implemented by RAT1:**procedure**
Man-in-the-middle Attack2:State ← {*mirror*, *send_to_position*, *circulate*, *fake_position*, *availability_attack*}3:    **Sniff packet**4:    **if**
*packet from client to robot*
**then**5:        **if State** is mirror **then**6:           **if**
*“command is turn right packet”*
**then**7:               *replace the TCP.payload by “turn left” payload*.8:           **if**
*“command is turn left packet”*
**then**9:               *replace the TCP.payload by “turn right” payload*.10:        **if State** is send_to_position **then**11:           replace the TCP.payload by “Go to X, Y” payload; the robot will go to specific location12:        **if State** is circulate **then**13:           replace the TCP.payload by “turn left” payload; the robot will turn around itself14:        **if State** is availability_attack **then**15:           drop the packet like if the robot is not present16:    **if**
*packet from robot to client*
**then**17:        **if State** is availability_attack **then**18:           drop the packet like if the robot is not present19:        **if State** is fake_position **then**20:           replace the TCP.payload with one with fake X,Y;21:    **close**;


#### 4.1.3. Analysis of Confidentiality Attacks

The last security objective that is affected is confidentiality. Utilizing the fact that there is no end-to-end encryption of the information sent between the client and the robot, and through a successful MITM attack, the attacker can see all the traffic in plain-text including username and password if security configuration is not enabled on the server. As such, confidentiality is a serious security flaw that has to be carefully addressed when using the robot’s software. For example, consider the case when the intercepted data could be a classified map of sensitive indoor facilities. Clearly, there will be devastating and harmful consequences.

### 4.2. Qualitative Assessment Results

Along with the risk assessment, it is important to conduct a qualitative assessment. In this type of assessment, we assign one of three levels (i.e., low, moderate or high) for the likelihood and the impact of an attack. In this subsection, we discuss the risk assessment table generation, which summarizes the assessment process. The adversarial risk assessment template proposed in [48] is used. Table 1 presents the end result of the risk assessment process for the threats under study. Following, each item in Table 1 is discussed (for more details about those items, the reader is advised to refer to [48]):

1. Threat Event: Refers to the threat that is currently being analyzed.
2. Threat Sources: Refer to the possible origins of the analyzed threat. According to [48], threat sources are classified into four categories: insiders, outsiders, trusted insiders, and privileged insiders. The threats considered in this paper require the attacker to be on the same network of the robot and the client. Hence, the attacker might be an insider or an outsider who hacked into the network. For example, in the case of a wireless network, an attacker can crack the WiFi password and get access to the network through many ways. The following three items are characteristics for the threat source.
3. Capability: This is the first characteristic of the threat source. An attacker with high capability is one who is well resourced and has high level of expertise. Due to the increasing interest in cyber-security, the number of highly capable threat sources is increasing, especially in scientific and research environments. The reader should note that integrity and availability attacks considered in this work, require traffic analysis and hacking skills. In addition, those attacks (except TCP SYN flood availability attack) target the application level of the client/robot connection, which requires the attacker to be knowledgeable in this area as well. Hence, the capability level of the threat source is ranked as high for all considered attacks except TCP SYN flood which can be initiated with moderate capability.
4. Intent: This is the second characteristic of the threat source. For the considered attacks, the adversary seeks to undermine critical functions of the system, which may result in physical damage by causing loss of integrity or availability.
5. Targeting: This is the third characteristic of the threat source. For the considered attacks, the adversary targets a specific mission or function for the robot or the user (i.e., the target of the attack is not random).
6. Relevance: This indicates how related the threat event is to the system under study. The relevance level could be one of the following: confirmed, expected, anticipated, predicted, possible, and not applicable. For example, if the threat event has already been observed in the system, then it is confirmed. Due to the lack of any evidence indicating that the considered threats happened on robot, we rank them as possible.
7. Likelihood of attack initiation: Refers to the probability that the adversary will initiate the attack. All threat events are ranked as moderate.
8. Severity of vulnerabilities: The analyzed integrity threat events leverage vulnerabilities in the data-link layer (through ARP spoofing) and the application layer in the communication link between the robot and the user. The same is true for the analyzed availability threat events that are based on MITM attack. On the other hand, the TCP SYN flood availability threat exploits vulnerabilities in the transport layer. According to the exposure, ease of exploitation, and the implemented security remediation against these vulnerabilities, we rank their severity as moderate.
9. Pervasiveness of predisposing conditions: The main predisposing condition of all considered threat events is for the attacker to be connected to the same network of victims (e.g., connect physically, crack WiFi password or be an insider). Since this predisposing condition applies to a major part of the system under attack, we rank its pervasiveness as moderate.
10. Likelihood initiated attack succeeds: Once the considered attacks are initiated, then it is almost certain that the integrity or the availability of the connection between the robot and the user will be lost. In this case, physical damage or human injury are somewhat likely.
11. Overall likelihood: This is a combination of the likelihood of attack initiation and the likelihood initiated attack succeeds. If both items are high, then the overall likelihood is high. If one of them is moderate and the other one is high, then the overall likelihood is moderate.
12. Level of impact: Severe or catastrophic effect on the target system means high impact. There is a very high possibility of physical damage/human injury for the considered attacks.
13. Risk: This is the final risk assessment measure, which is the product of the overall likelihood and the level of impact. Moderate likelihood and high impact result in moderate risk [48].

### 4.3. Cyber-Physical Consequences

The cyber-physical impacts of the various security attacks described previously depend on the application that the robot is running and its associated sensors. To illustrate the severity of the cyber-physical consequences, we consider the semi-autonomous mapping task using the PeopleBot robot, as shown in Figure 1. We desire to map the indoor hallways of the second floor of the engineering building at our University. The robot is remotely teleoperated using MobileEyes and arnlServer. MobileEyes allows the robot to be teleoperated in the indoor hallways, while being fully monitored using its PTZ camera located on top of the 2D lidar sensor. During the robot operation, the 2D lidar data and the wheel encoders of the robot are logged into the memory of the robot’s onboard computer to be processed later to create the map of the environment. All indoor hallways are fully covered with the university WiFi signal. This insures that the robot will have full coverage to all indoor hallways while being teleoperated without any concern of being disconnected from the network. The generated map will be used to navigate the robot autonomously within the hallways to perform certain tasks, such as delivering mail to various departments in the engineering college.

**Figure 8:**
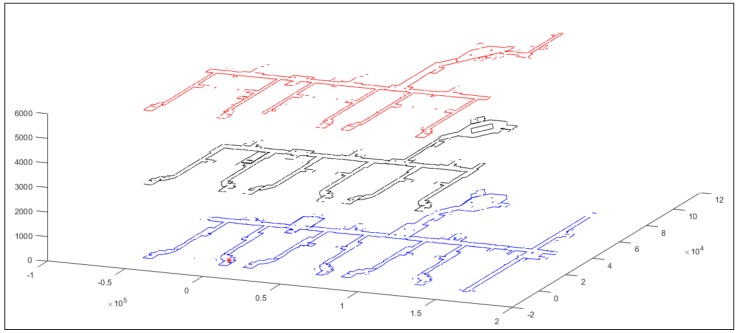
2D line map of the indoor hallways in three floors (first, second and third) of the engineering building at our university. Units are in mm. The map in blue, black, and red colors are for the first, second and third floors, respectively. The position of the red asterisk represents the (0, 0, 0) coordinate.

Figure 8 shows the created map of the engineering building covering the three floors of the building. It is important to mention that, in other applications, the controlled robot might be at an assembly line, engaged in a surgical procedure, or performing a time-sensitive military task. As such, again, the physical consequences of any cyber-physical attacks on these applications can result in catastrophic outcomes. While teleoperating the robot using MobileEyes, several attacks from the attack trees in Figure 6 and Figure 7 are performed and their impacts on integrity, availability, and confidentiality are observed. In the following, we present a short list of some of the possible physical consequences or impacts that can result from such attacks:

- Integrity attacks can result in:
  - Manipulating the behavior of the robot;
  - Delaying or preventing the robot from performing its assigned task, which can be very time sensitive (e.g., delivering urgent mail);
  - Hijacking the robot physically;
  - Navigating the robot to unintended or unauthorized place(s);
  - Denying remote access service to the robot, where the robot becomes unavailable;
  - Colliding with humans or equipments, which could result in injuries and damages;
  - Stealing or hijacking sensitive information on the robot; and
  - Limiting or reducing certain functions on the robot.
- Availability attacks can result in:
  - Denying remote access service to the robot;
  - Having the robot stolen;
  - Having the robot physically damaged; and
  - Colliding with humans or equipments, which could result in injuries and damages.
- Confidentiality attacks can result in:
  - Having sensitive information on the robot being hijacked and/or stolen.

We should mention that we also implemented the same attacks on various simulated Adept mobile robots using MobileSim and the results were similar to the ones we got from attacking the real robot (i.e., PeopleBot). There are no differences between the results obtained from MobileSim and the real robot. In fact, our approach does not really discriminate between the two sets of experiments. We used MobileSim simulator to show that the attacks we successfully performed on the PeopleBot also applies to the rest of the robotic platforms from Adept company. Additionally, it is important to mention that the main components in the underlying robotic system to be attacked and analyzed are: the client (MobileEyes), the server (arnlServer), and the robot. In the case the robot is physically available, the server needs to run on the robot’s main computer. However, if the robot is not physically available, MobileSim is used to only act as a virtual robot. In this case, the server needs to run on the same machine on which MobileSim runs.

It is clear that the physical consequences described above are potentially extensive and of high risk. As such, one needs to carefully consider taking well-studied countermeasures to reduce or even prevent them. The next subsection suggests some mitigation techniques.

### 4.4. Suggested Mitigation Techniques

To remedy the moderate risk levels and the severe physical consequences associated with the security threats assessed in the previous subsections, appropriate mitigation techniques must be deployed in the system. For a mitigation technique to be effective against a specific security threat, the vulnerabilities exploited by the threat (e.g., vulnerabilities found in the robot’s software as listed in Section 3) must be taken into consideration.

The assessed integrity threats involve manipulating the bidirectional data communicated between the client and the robot. An effective approach to overcome these threats is to provide an end-to-end encryption of the traffic (e.g., authentication information, robot position, map of the environment, and commands) between the client and the robot. Such mitigation technique should take into consideration the time sensitivity of the robot applications, which might get impacted by the encryption overhead. In time-critical robot tasks, designers could consider lightweight encryptions instead of conventional encryptions because lightweight encryptions have less overhead on system resources [58].

Regarding the availability threat, which is based on corrupting the configuration files, there is a mitigation technique with two requirements to reduce the possibility of the loss of availability. The first requirement is to replace the vulnerable MD5 hashing algorithm used for authenticating the password between client and server with a better hashing algorithm. The second requirement is to set “user login” as a default prerequisite for configuring the server (i.e., the robot).

To mitigate the availability threat, which involves dropping certain packets, one can enforce disconnection and timeout policies whenever the client is disconnected from the server for higher than a specific threshold period of time. Such technique can help to discover MITM attack; however, there is a chance that time sensitive applications will be affected, which must be carefully investigated.

In addition, there are various generic countermeasures, which can limit the negative impacts of different security threats on the robotic platforms. For example, the robot can be equipped with an intrusion detection system (IDS) or one of the known techniques to prevent man-in-the-middle attacks and/or attacks of similar kind. Another example is improving network security to deny unauthorized access to the robot network. For all such generic mitigation techniques, it is important to assess the overhead introduced by embedding the new security enforcement, which is left for future work.

Lastly, there is a great necessity to raise awareness of the importance of deploying effective security countermeasures and mitigation techniques in the robotic platforms through seminars, workshops, presentations, and others.

## 5. Conclusions and Future Directions

In this paper, we analyze the cyber-physical security threats, which target the communication link between Adept MobileRobots platforms and their clients. Initially, we analyze vulnerabilities in the communication link and in MobileEyes/arnlServer robotic applications. Next, we target three major security requirements, integrity, availability, and confidentiality, using an impact-oriented approach. We followed the systematic methodology for risk assessment proposed in NIST adversarial risk assessment template [48]. We designed a novel robot attack tool (RAT) to mount certain security attacks. Our risk assessment was evaluated using simulation-based approach through MobileSim simulator, and physically on the PeopleBot robotic platform. The risk level of the performed attacks were qualitatively assessed, possible physical consequences were identified, and some mitigation techniques to reduce or prevent the risk of attacks were suggested. Our goal was to improve safety and security of the robotic platforms, raise awareness and increase understanding of the emerging threats on the robotic platforms.

Future work can study and analyze threats in other robotic platforms and applications. Another important research direction is to study the overhead introduced by embedding new security countermeasures (e.g., IDS) on the system resources and on the real time sensitive applications. Finally, employing lightweight encryption techniques between client and robot is another interesting point to study.

## Figures and Tables

**Table 1:** Risk assessment table.

1	2	3	4	5	6	7	8	9	10	11	12	13
Threat Event	Threat Sources	Threat Source Characteristics			Relevance	Likelihood of Attack Initiation	Severity of Vulnerabilities	Pervasiveness of Predisposing Conditions	Likelihood Initiated Attack Succeeds	Overall Likelihood	Level of Impact	Risk
		Capability	Intent	Targeting								
Manipulate user commands
(Cyber-physical Attack).	Insider or outsider	High	High	High	Possible	Moderate	Moderate	Moderate	High (loss of integrity)	Moderate	High	Moderate
Manipulate data and feedback from the robot (Cyber-physical Attack)	Insider or outsider	High	High	High	Possible	Moderate	Moderate	Moderate	High (loss of integrity)	Moderate	High	Moderate
TCP SYN flood on port 7272	Insider or outsider	Moderate	High	High	Possible	Moderate	Moderate	Moderate	High (loss of availability)	Moderate	High	Moderate
Corrupt configuration files on robot or client	Insider or outsider	High	High	High	Possible	Moderate	Moderate	Moderate	High (loss of availability)	Moderate	High	Moderate
Drop certain commands so that certain functions are not available	Insider or outsider	High	High	High	Possible	Moderate	Moderate	Moderate	High (loss of availability)	Moderate	High	Moderate
